# A survey of undernutrition in children under three years of age in rural Western China

**DOI:** 10.1186/1471-2458-14-121

**Published:** 2014-02-05

**Authors:** Leilei Pei, Lin Ren, Hong Yan

**Affiliations:** 1 Department of Epidemiology and Health Statistics, School of Public Health, Xi’an Jiaotong University Health Science Center, Xi’an, Shaanxi 710061, P.R. China; 2 Department of Quality Control, Xi’an No.4 hospital, Xi’an, Shaanxi 710004, P.R. China; 3 Nutrition and Food Safety Engineering Research Center of Shaanxi Province, Xi’an, Shaanxi 710061, P.R. China

**Keywords:** Children, Undernutrition, Western China

## Abstract

**Background:**

Childhood undernutrition adversely impacts child health and is one of China’s largest health burdens. However, there is limited information on the current rate of childhood undernutrition in rural Western China. The purpose of this study was to investigate the prevalence of childhood undernutrition and explore its association with socio-economic characteristics in Western China.

**Methods:**

A total of 13,532 children of 0 ~ 36 months of age were recruited as subjects from 45 counties and 10 provinces in Western China with a 3-stage probability proportion to size sampling. The composite index of anthropometric failure (CIAF) was used to assess the childhood undernutrition. The association between socio-economic characteristics and childhood undernutrition was analyzed using a two-level logistic regression.

**Results:**

Based on CIAF, the prevalence of undernutrition among children under three years of age in rural Western China in 2005 was 21.7%. The two-level logistic analysis presented a large difference in undernutrition among the 10 provinces with the highest odds ratio in Guizhou (OR: 2.15, 95%CI: 1.50, 3.08). Older children had a higher prevalence of undernutrition. As compared to girls, boys were more likely to be undernourished (OR 1.27, 95% CI: 1.16, 1.39). The likelihood of undernutrition was lower in subjects of Han ethnicity as opposed to subjects of minority ethnicities (OR 0.77, 95%CI: 0.65, 0.90). In addition, the education levels of the mother as well as wealth index were both negatively associated with childhood undernutrition.

**Conclusions:**

Childhood undernutrition still remains a large health challenge in rural Western China. This study has important policy implications for the Chinese government to improve childhood undernutrition in the surveyed areas.

## Background

Undernutrition commonly encompasses stunting, wasting, as well as deficiencies of essential vitamins and minerals [1]. Child undernutrition is a worldwide health concern, which often causes irreversible damage to the physical and mental health of children as well as also adversely affects the health and productivity throughout adulthood [2]. According to data from World Health Organization (WHO), it is estimated that 60% of the 10.9 million annual deaths among children younger than five years old in low-income and middle-income countries are directly associated with undernutrition [3]. Moreover, child undernutrition is an important indicator of the Millennium Development Goals (MDG) set between 1990 and 2015, which aim to halve the proportion of people who suffer from hunger [4].

Western China consists of 12 provinces and has a total land area of 5.38 million square kilometers, a population of 287 million and 44 ethnicities. Due to lower socio-economic status and inadequate health facilities, Western China may face a severe challenge of childhood undernutrition. A 1999 survey in rural Western China indicated approximately 23 percent of the children under 3 years old were stunted, 7.5% wasted and 22.6% underweight [5]. With the exception of the study mentioned above, there remains limited updated information on the rate of child undernutrition in Western China. Hence in 2005, a large-scale sampling health survey directed at children under three years old sponsored by the United Nations Children’s Fund (UNICEF) and Chinese Ministry of Health (MOH) was carried out in rural Western China. The primary goal of our study was to investigate the prevalence of child undernutrition and explore its association with socio-economic characteristics.

## Methods

### Study design

According to the cross-sectional design of the study, a three-stage sampling method was used to select children under three years old in 45 counties within 10 provinces in Western China from June to August in 2005. Sampling procedures are presented in detail as follows. First, 45 counties within 10 provinces were determined by the Chinese Ministry of Health (MOH) and the United Nations Children’s Fund (UNICEF). Secondly, five townships were selected from each selected county with the probability proportional to the total population (from China’s demographic data). In each selected township, four villages were also chosen with the probability proportional to the population size. Finally, 16 children less than three years old were selected randomly in each chosen village.

### Human subjects and data collection

Before the survey, informed consents were first obtained from the mothers of children. Then, a face-to-face interview was conducted with a family questionnaire to collect the child’s information and socio-demographic characteristics. The family questionnaires used for data collection had a total of 98 questions and was grouped into five parts, which include: 1) general information on the family, 2) health care practices and occurrence of diseases (including childhood cold and diarrhea), 3) maternal health care and pregnancy care, 4) anthropometrics and 5) cooperative medical treatment. For anthropometrics, both height and weight measurements of children were recorded by standard methods using calibrated instruments in an empty room in every sampled village. Overall field surveys and data collection were carried out by the trained field staff from Xi’an Jiaotong University College of Medicine. The whole study was reviewed and approved by the Human Research Ethics Committee of the Xi’an Jiaotong University College of Medicine.

### Definitions of indicators

In the study, breastfeeding denotes that children ever had mother’s milk after birth. The percentage of breastfed children who had ever had mother’s milk were accounted for in the population of surveyed children. Currently breastfeeding indicates that the child still feeds from the mother’s milk at the time of the survey.

Anthropometric indicators are calculated from the age, height and weight. These indicators include the height-for-age (HA), weight-for-height (WH) and weight-for-age (WA), which are often employed to assess a child’s nutritional status. These measures are expressed in the form of a Z-score, which include HAZ, WHZ and WAZ, according to the new WHO child growth standards released in April 2006 [6]. For example the height-for-age Z-score of child “*i*” is given as:

(1) $$\;Z‒\text{score}=\frac{H_{i}-H_{\text{reference}}}{SD_{\text{reference}}}$$

Where, H_i_ and *H*_*reference*_ are the height of the surveyed child and the median height of the reference population respectively; *SD*_*reference*_ is the standard deviation of the reference population height.

Generally speaking, stunting (HAZ < −2) represents past or chronic malnutrition or illness, but is less sensitive to temporary food shortages. Wasting (WHZ < −2) is sensitive to temporary food shortages and episodes of illness and commonly used as an indicator of current nutritional status [7]. In addition, underweight (WAZ < −2) reflects the cumulative and acute exposures [8].

Studies showed that the three conventional indicators only provided the categorization of children into the general categories of undernutrition and did not determine the overall prevalence of undernutrition associated with multiple failures [9-11]. Consequently, these indicators are not sufficient and may underestimate the prevalence of undernutrtion due to the potential overlap of children into multiple categories of anthropometric failure. As a result, in this study we applied another alternative indicator of malnutrition–the composite index of anthropometric failure (CIAF) to assess childhood undernutrition [10].

On the basis of the CIAF, children are categorized into seven groups. “No failure” is defined as children not suffering from any anthropometric failure. “Wasting only” is defined as children with acceptable WA and HA but who have subnormal WH. “Wasting and underweight” are children with acceptable HA but low WA and WH. “Wasting, stunting and underweight” are children who suffer from anthropometric failure on all three measures. “Stunting and underweight” are children with low WA and low HA but acceptable WH. “Stunting only” are children with low HA but acceptable WA and WH. “Underweight only” are children who have low WA only [9]. Thus, the composite index of anthropometric failure (CIAF) can be defined as children suffering from any anthropometric failure above.

### Statistical analysis

Child undernutrition based on CIAF was treated as the sole outcome variable in multivariate analysis. Considering the hierarchical structure of the sampling strategy, a multi-level logistic model was applied to assess the association between child undernutrition and explanatory variables. Data was entered twice into Info Version 6.0 software (CDC, Atlanta, GA, USA) and all statistical analysis was performed using STATA version 12.0 software (STATA Corporation, College Station, TX, USA).

#### Explanatory variables

In this study, explanatory variables included: (i) parity (continuous) which refers to the number of times a mother has given birth to a baby, (ii) child gender (boy or girl), (iii) ethnicity (Han or Minority), (iv) currently breast-feeding (breastfed or not breastfed), (v) child’s age in months (0 ~ 6, 7 ~ 12, 13 ~ 18, 19 ~ 24, 25 ~ 30, and 31 ~ 36 months groups), (vi) mothers’ age (<20, 20 ~ 29, 30 ~ 39, and 40 ~ years groups) and (vii) mother’s education in year (no education, 1 ~ 5, 6 ~ 9, 10 ~ 12 and >12 years).

On account of lack of information on household income, a wealth index developed by means of the principle component analysis (PCA) was used as a proxy measure for the economic status of the households. The index combined information on an inventory of household assets or facilities, including five variables such as: the resources of household income, type of television, type of vehicle, texture of pot and the availability of clean water. In this process, three principal components with eigenvalue over one were extracted, but only the first of the components produced with maximum variance was used to represent the household wealth. Based on the tertiles of the first principal component, the population was categorized into three categories indicating the poor, middle and the rich [12,13].

When assessing the differences between 10 provinces, effect coding was introduced in the study. Effect coding uses only ones, zeros and minus ones to convey all of the necessary information on group membership. In general, with k groups there will be k-1 coded variables. For this study in which there are 10 provinces we will need to create 9 effect coded variables. The following process is how effect variables were created, which we represented as e1, e2, e3,…,e9. For e1, every observation in province 1 will be coded as 1 and 0 for provinces 2, 3,…,9, and −1 for province 10. We then code e2 with 1 if the observation is in province 2 and zero for provinces 1, 3,…,9, and −1 for province 10. Analogically, e3,…,e9 were also coded with the same method. The group with all -1 s is known as the reference group, which in our example is province 10.

For regression analysis with effect coding, the constant is equal to the grand mean of all of the observations. The coefficients of the effect variables is equal to the difference between the mean of the group coded 1 and the grand mean. If province 1 was chosen as the reference group, coded with all -1 s and introduced into regression analysis once again, the value of the constant would still be the grand mean. The coefficients for e2-e9 will have the same values, but the coefficients for e1 will be replaced by the coefficient for e10, the difference between the grand mean and province 10. In this case, we can obtain the differences among 10 provinces with the grand mean of the 10 provinces as the reference category.

#### Two-level logistic regression

The observed responses *y*_*ij*_ are proportions with the standard assumption that they are binomially distributed *y*_*ij*_ = *Bin*(*π*_*ij*_, *n*_*ij*_), where *n*_*ij*_ is the denominator for the proportion. A random intercept model corrects for the problem of correlated observations in a cluster by introducing a random effect at each cluster. Consider the two-level logistic random intercept model where the expected proportion is modeled using a logit link function.

(2) $$P_{\text{ij}}={\left\{1+\exp\left(-\left[\beta_{0j}+\beta_{1}X_{1\text{ij}}+\cdots +\beta_{m}X_{\text{mij}}\right]\;\right)\right\}}^{-1}$$

Where, *β*_0*j*_ = *β*_0_ + *u*_0*j*_, $u_{0j}\sim \left(0,\sigma_{u_{0}}^{2}\right)$, var(*P*_*ij*_) = *δπ*_*ij*_(1 − *π*_*ij*_)/*n*_*ij*_, *δ* was scale parameter. The model could also be expressed as follows,

(3) $$\log\text{it}\left(P_{\text{ij}}\right)=\beta_{0}+\beta_{1}X_{1\text{ij}}+\cdots +\beta_{m}X_{\text{mij}}+u_{0j}$$

First, we constructed a five-level logistic null model representing province-county-township-village-individual and found variances that the random effects in each level were 0.34, 0.58, 0.00 and 0.33, respectively, with the highest at the county level. For result interpretation, we utilized a simplified logistic random intercept model that adopted two levels, in which level 1 represented the 13532 individuals and level 2 was the 45 counties. *P*_*ij*_ was the proportion of undernutrition from the j^th^ county in the i^th^ individual. *β*_1_ ⋯ *β*_*m*_ were the regression coefficients corresponding to the effects of fixed covariates, *X*_1*ij*_ ⋯ *X*_*mij*_ were the explanatory variables with observed characteristics of the children and mothers. In order to test differences in childhood undernutrition among 10 provinces after controlling for other socio-economic characteristics, the effect coded variables of the 10 provinces were also included in the individual level. The random effects were presented as random variance with standard error (SE) and the fixed effects were represented as an odds ratio (OR) with 95% confidence interval (CI). Taking into account that the variances at individual and area level were not directly comparable in the two-level logistic regression, variance partition coefficient (VPC) was calculated by means of latent variable method as the proportion of variation that is attributable to the county-level sources. Besides, median odd ratios (MOR) and proportional change in variance (PCV) were also obtained to estimate the area level and individual level variation respectively [14,15].

## Results

### Baseline characteristics

A total of 894 villages were sampled out of 225 townships and 14,112 children were involved. Five hundred and eighty children were excluded from the study for several reasons, which included children or their parents being unavailable for measurements, children birth dates being unknown and children having improbable Z-scores or exceeding the ranges (HAZ: <−6 or >6; WAZ: <−6 or >5; WHZ: <−5 or >5) [16]. As a result, a total of 13,532 children of 0 ~ 36 months of age were enrolled. Of the participants, 63.8% were from the Han ethnic group and boys accounted for 57.5%. The average age of children was 17.3 ± 9.7 months. The mean years of the mothers’ education was 6.5 ± 3.2 years (range 0–23 years), and the average maternal age was 26.7 ± 4.7 years (range 15–53 years). The percentage of breastfed children reached 95.6% in 2005. The wealth index ranged within three levels and included 27.9% of children from poor households, 37.0% from middle households and 35.1% from rich households (Table 1). The statistical comparisons of baseline characteristics of the children and their mothers between provinces are shown in Table 1. We show that there are statistically significant differences in the children’s age, gender, ethnicity, currently breast-fed status of child and mothers’ age, mothers’ education years as well as wealth index.

**Table 1:** **Baseline characteristics of children under three years old in 2005 in 10 provinces**^**a**^

**Characteristics**^**b**^	**Gansu**	**Guangxi**	**Guizhou**	**Jiangxi**	**Inner Mongolia**	**Ningxia**	**Qinghai**	**Sichuan**	**Xinjiang**	**Chongqing**
Parity†	1.6 ± 0.8	1.4 ± 0.6	1.5 ± 0.7	1.5 ± 0.6	1.2 ± 0.4	1.5 ± 0.7	1.4 ± 0.6	1.5 ± 0.5	1.6 ± 0.8	1.3 ± 0.5
Gender*										
Girl	138(44.8)	660(41.8)	480(38.2)	483(31.0)	550(45.3)	505(40.2)	742(47.0)	668(42.7)	1008(47.1)	516(47.8)
Boy	170(55.2)	919(58.2)	776(61.8)	1073(69.0)	663(54.7)	752(59.8)	837(53.0)	895(57.3)	1133(52.9)	564(52.2)
Ethnicity*										
Minority	3(1.0)	999(63.3)	896(71.3)	11(0.7)	66(5.4)	232(18.5)	586(37.1)	529(33.8)	1727(80.7)	24(2.2)
Han	305(99.0)	580(36.7)	360(28.7)	1545(99.3)	1147(94.6)	1025(81.5)	993(62.9)	1034(66.2)	414(19.3)	1056(97.8)
Currently breast-feeding*										
No	13(4.2)	32(2.0)	64(5.1)	84(5.4)	70(5.8)	61(4.9)	46(2.9)	84(5.4)	106(5.0)	29(2.7)
Yes	295(95.8)	1538(98.0)	1188(94.9)	1471(94.6)	1143(94.2)	1193(95.1)	1532(97.1)	1479(94.6)	2034(95.0)	1050(97.3)
Children' age (months)‡										
0 ~ 6	23(7.5)	283(17.9)	143(11.4)	274(17.6)	191(15.7)	148(11.8)	145(9.2)	191(12.2)	331(15.5)	120(11.1)
7 ~ 12	51(16.6)	408(25.8)	270(21.5)	366(23.5)	336(27.7)	286(22.8)	378(23.9)	314(20.1)	471(22.0)	216(20.0)
13 ~ 18	52(16.9)	326(20.6)	220(17.5)	302(19.4)	236(19.5)	235(18.7)	294(18.6)	293(18.7)	389(18.2)	192(17.8)
19 ~ 24	64(20.8)	271(17.2)	262(20.9)	303(19.5)	178(14.7)	220(17.5)	278(17.6)	283(18.1)	343(16.0)	192(17.8)
25 ~ 30	47(15.3)	150(9.5)	175(13.9)	168(10.8)	99(8.2)	168(13.4)	234(14.8)	229(14.7)	311(14.5)	161(14.9)
31 ~ 36	71(23.1)	141(8.9)	186(14.8)	143(9.2)	173(14.3)	200(15.9)	250(15.8)	253(16.2)	296(13.8)	199(18.4)
Mothers’ age‡										
<20	2(0.7)	17(1.1)	23(1.9)	23(1.5)	12(1.0)	26(2.1)	79(5.0)	29(1.9)	113(5.3)	12(1.2)
20 ~ 29	223(72.6)	1017(64.5)	900(73.5)	1122(73.4)	950(78.6)	962(78.7)	1153(73.6)	976(63.2)	1439(67.7)	635(65.4)
30 ~ 39	80(26.1)	514(32.6)	286(23.4)	378(24.7)	242(20.0)	229(18.7)	328(20.9)	505(32.7)	548(25.8)	298(30.7)
40~	2(0.7)	28(1.8)	15(1.2)	6(0.4)	5(0.4)	5(0.4)	6(0.4)	35(2.3)	26(1.2)	26(2.7)
Mothers’ educations years‡										
no education	83(27.1)	46(2.9)	197(15.7)	74(4.8)	76(6.3)	155(12.5)	428(27.3)	83(5.3)	72(3.4)	20(1.9)
1 ~ 5	106(34.6)	484(30.7)	364(29.1)	662(43.1)	186(15.3)	245(19.8)	400(25.5)	269(17.3)	713(33.3)	50(4.7)
6 ~ 9	94(30.7)	941(59.6)	644(51.4)	729(47.4)	772(63.6)	731(59.1)	563(36.0)	1103(70.8)	1182(55.3)	848(80.5)
10 ~ 12	15(4.9)	80(5.1)	40(3.2)	58(3.8)	134(11.0)	86(7.0)	120(7.7)	79(5.1)	143(6.7)	80(7.6)
>12	8(2.6)	28(1.8)	8(0.6)	14(0.9)	45(3.7)	19(1.5)	55(3.5)	25(1.6)	29(1.4)	55(5.2)
Wealth index‡										
Poor	79(25.6)	336(21.3)	559(44.5)	222(14.3)	161(13.3)	100(8.0)	683(43.3)	282(18.0)	1281(59.8)	130(12.0)
Middle	53(17.2)	639(40.5)	353(28.1)	512(32.9)	430(35.4)	418(33.3)	587(37.2)	823(52.7)	658(30.7)	564(52.2)
Rich	176(57.1)	604(38.3)	344(27.4)	822(52.8)	622(51.3)	739(58.8)	309(19.6)	458(29.3)	202(9.4)	386(35.7)
Total	308	1579	1256	1556	1213	1257	1579	1563	2141	1080

^a^Values are expressed as mean ± SD or the percentage of the study population in that province.

^b^Missing values: 3 for parity, 21 for breastfeeding, 3 for gender, 92 for mothers’ education years, 262 for mothers’ age.

†denote *P <* 0.05, suggesting a significant difference in parity among 10 provinces by using one-way ANOVA; *denote *P <* 0.05 in gender, ethnicity and currently breast-feeding among the 10 provinces with *χ*^2^ test; ‡ denote *P <* 0.05 in children’ age, mothers’ educations years and wealth index among the 10 provinecs by Kruskal-Wallis H test.

### Prevalence of undernutrition

Table 2 presents the prevalence of child undernutrition based on different classifications of CIAF across 10 provinces in rural Western China in 2005. Amongst all of the children, the prevalence of “stunting only” was the highest (11.6%), followed by “underweight and stunting” (4.0%), “wasting and underweight” (2.2%), “underweight only” (2.1%), “wasting only” (2.1%) and “wasting, stunting and underweight” (0.9%). The overall rate of child undernutrition based on the CIAF was 21.7% in surveyed areas in 2005. Among the 10 provinces, the prevalence of child undernutrition was the highest in Guizhou (39.3%). Across different child and mothers’ characteristics, the differences in the prevalence of all categories of CIAF were statistically significant. The rates of “stunting only” and “wasting, stunting and underweight” were much higher among boys than girls (*P <* 0.05). Children of Minority ethnicities were more likely to suffer from undernutrition in different classifications in contrast to Han children (*P <* 0.05). “Wasting only”, “wasting and underweight” and “wasting, stunting and underweight” were more likely to occur among children less than 18 months, but the rates of “stunting only” and “underweight and stunting” were higher in children aged 25 months and above (*P <* 0.05). It was also found that children of mothers with education of 12 years and above were less likely to become undernourished (*P <* 0.05). Furthermore, children from wealthier households had the lowest rates of undernutrition in different classifications (*P <* 0.05).

**Table 2:** **Prevalence of undernutrition in children aged 0–36 months based on the different classifications across baseline characteristics**^**a**^

**Characteristics**	**CIAF**	**No failure**	**Wasting only**	**Wasting and underweight**	**Wasting,stunting and underweight**	**Underweight and stunting**	**Stunting only**	**Underweight only**
	**NO. (%)**	**NO. (%)**	**NO. (%)**	**NO. (%)**	**NO. (%)**	**NO. (%)**	**NO. (%)**	**NO. (%)**
Province								
Gansu	74(24.0)*	234(76.0)*	6(1.9)*	4(1.3)*	2(0.6)*	13(4.2)*	49(15.9)*	0(0.0)*
Guangxi	395(25.0)	1184(75.0)	43(2.7)	48(3.0)	28(1.8)	77(4.9)	175(11.1)	21(1.3)
Guizhou	494(39.3)	762(60.7)	34(2.7)	54(4.3)	28(2.2)	135(10.7)	210(16.7)	28(2.2)
Jiangxi	298(19.2)	1258(80.8)	37(2.4)	41(2.6)	15(1.0)	46(3.0)	142(9.1)	17(1.1)
Inner Mongolia	95(7.8)	1118(92.2)	15(1.2)	5(0.4)	2(0.2)	12(1.0)	56(4.6)	5(0.4)
Ningxia	135(10.7)	1122(89.3)	26(2.1)	18(1.4)	6(0.5)	20(1.6)	60(4.8)	5(0.4)
Qinghai	295(18.7)	1284(81.3)	20(1.3)	25(1.6)	4(0.3)	39(2.5)	199(12.6)	4(0.3)
Sichuan	472(30.2)	1091(69.8)	53(3.4)	51(3.3)	16(1.0)	77(4.9)	265(17.0)	8(0.5)
Xinjiang	429(20.3)	1712(80.0)	32(1.5)	33(1.5)	21(1.0)	76(3.5)	244(11.4)	21(1.0)
Chongqing	247(22.9)	833(77.1)	12(1.1)	12(1.1)	5(0.5)	40(3.7)	170(15.7)	3(0.3)
Gender								
Girl	1119(19.5)*	4631(80.5)	106(1.8)	117(2.0)	35(0.6)*	223(3.9)	591(10.3)*	39(0.7)
Boy	1815(23.3)	5967(76.7)	172(2.2)	174(2.2)	92(1.2)	312(4.0)	979(12.6)	73(0.9)
Ethnicity								
Minority	1410(27.8)*	3663(72.2)	106(2.1)	129(2.5)*	70(1.4)*	300(5.9)*	734(14.5)*	61(1.2)*
Han	1524(18.0)	6935(82.0)	172(2.0)	162(1.9)	57(0.7)	235(2.8)	836(9.9)	51(0.6)
Currently breast-feeding								
No	129(21.9)	460(78.1)	6(1.0)	13(2.2)	7(1.2)	27(4.6)	72(12.2)	3(0.5)
Yes	2799(21.7)	10124(78.3)	272(2.1)	278(2.2)	120(0.9)	507(3.9)	1493(11.6)	109(0.8)
Children’ age (months)								
0 ~ 6	245(13.3)	1604(86.7)	71(3.8)*	28(1.5)*	2(0.1)*	23(1.2)*	112(6.1)*	8(0.4)*
7 ~ 12	490(15.8)	2606(84.2)	72(2.3)	82(2.6)	23(0.7)	68(2.2)	209(6.8)	33(1.1)
13 ~ 18	544(21.4)	1995(78.6)	47(1.9)	69(2.7)	43(1.7)	84(3.3)	277(10.9)	19(0.7)
19 ~ 24	639(26.7)	1755(73.3)	33(1.4)	40(1.7)	32(1.3)	117(4.9)	390(16.3)	22(0.9)
25 ~ 30	467(26.8)	1275(73.2)	25(1.4)	32(1.8)	14(0.8)	120(6.9)	259(14.9)	16(0.9)
31 ~ 36	549(28.7)	1363(71.3)	30(1.6)	40(2.1)	13(0.7)	123(6.4)	323(16.9)	14(0.7)
Mothers’ age								
<20	69(20.5)	267(79.5)	8(2.4)	6(1.8)	2(0.6)	14(4.2)	35(10.4)	4(1.2)
20 ~ 29	2007(21.4)	7370(78.6)	194(2.1)	212(2.3)	80(0.9)	349(3.7)	1077(11.5)	81(0.9)
30 ~ 39	776(22.8)	2632(77.2)	66(1.9)	68(2.0)	40(1.2)	150(4.4)	420(12.3)	25(0.7)
40~	33(21.4)	121(78.6)	6(3.9)	3(1.9)	1(0.6)	9(5.8)	12(7.8)	2(1.3)
Mothers’ educations years								
no education	340(27.6)*	894(72.4)	19(1.5)	28(2.3)	21(1.7)*	81(6.6)*	176(14.3)*	11(0.9)
1 ~ 5	904(26.0)	2575(74.0)	61(1.8)	86(2.5)	46(1.3)	184(5.3)	489(14.1)	33(0.9)
6 ~ 9	1543(20.3)	6064(79.7)	180(2.4)	162(2.1)	55(0.7)	244(3.2)	829(10.9)	61(0.8)
10 ~ 12	97(11.6)	738(88.4)	14(1.7)	11(1.3)	5(0.6)	15(1.8)	48(5.7)	4(0.5)
>12	36(12.6)	250(87.4)	4(1.4)	2(0.7)	0(0.0)	5(1.7)	22(7.7)	3(1.0)
Wealth index								
poor	1054(27.5)*	2779(72.5)	83(2.2)	97(2.5)	59(1.5)*	205(5.3)*	557(14.5)*	41(1.1)
middle	1046(20.8)	3991(79.2)	108(2.1)	106(2.1)	35(0.7)	190(3.8)	572(11.4)	32(0.6)
rich	834(17.9)	3828(82.1)	87(1.9)	88(1.9)	33(0.7)	140(3.0)	441(9.5)	39(0.8)
Total	2934(21.7)	10598(78.3)	278(2.1)	291(2.2)	127(0.9)	535(4.0)	1570(11.6)	112(2.1)

^a^Values represent the number of children with anthropometric failure and the prevalence is included in the bracket.

*denote *P <* 0.05 for prevalence of undernutrition based on the different classifications across baseline characteristics using *χ*^2^ test.

### Predictor of undernutrition

A two-level logistic random intercept regression analysis was performed to determine the contributing factors to childhood undernutrition (Table 3). Regarding the random effect, VPC decreased from 17.0% to 9.8% between the null model and the full model, suggesting that a cluster effect still existed at the county level after controlling for other factors. In the full model, the MOR was equal to 1.76, which suggested that children from two different counties will have a median difference in the odds of undernutrition by 1.76 times due to county-level heterogeneity. Comparing the null model with the full model, PCV was equal to 46.3%, reflecting that 46.3% of the area variance in the null model was attributable to the individual factors considered.

**Table 3:** Predictors of childhood undernutrition: results from two-level logistic regression analysis

**Characteristics**	**Null model**	**Random intercept model with individual variables**
*Fixed effects (OR, 95% CI)*		
Individual level variables		
Province		
Gansu		1.18 (0.70, 1.99)
Guangxi		1.25 (0.90, 1.74)
Guizhou		2.15 (1.50, 3.08)
Jiangxi		0.99 (0.71, 1.38)
Inner Mongolia		0.41 (0.28, 0.60)
Ningxia		0.49 (0.34, 0.72)
Qinghai		0.82 (0.59, 1.14)
Sichuan		1.77 (1.28, 2.45)
Xinjiang		0.79 (0.58, 1.06)
Chongqing		1.40 (0.97, 2.02)
Children’ age (months)		
0 ~ 6		1.00 (Reference)
7 ~ 12		1.27 (1.07, 1.50)
13 ~ 18		1.84 (1.55, 2.18)
19 ~ 24		2.41 (2.03, 2.86)
25 ~ 30		2.47 (2.06, 2.96)
31 ~ 36		2.86 (2.39, 3.41)
Parity		1.05 (0.97, 1.13)
Gender		
girl		1.00 (Reference)
boy		1.27 (1.16, 1.39)
Currently breastfeeding		
no		1.00 (Reference)
yes		0.94 (0.76, 1.17)
Ethnicity		
Minority		1.00 (Reference)
Han		0.77 (0.65, 0.90)
Wealth index		
poor		1.00 (Reference)
middle		0.86 (0.76, 0.97)
rich		0.79 (0.69, 0.90)
Mothers’ educations years		
no education		1.00 (Reference)
1 ~ 5		0.89 (0.76, 1.05)
6 ~ 9		0.75 (0.64, 0.89)
10 ~ 12		0.47 (0.36, 0.61)
>12		0.52 (0.34, 0.79)
Mothers’ age (years)		
<20		1.00 (Reference)
20 ~ 29		0.88 (0.66, 1.17)
30 ~ 39		0.81 (0.60, 1.10)
40~		0.65 (0.39, 1.07)
*Random effects*		
County level variance (SE)	0.67 (0.08)	0.36 (0.05)
VPC (latent variable method)	17.0%	9.8%
PCV	Reference	46.3%
MOR (95% CI)	2.18 (2.01,2.39)	1.76 (1.65, 1.90)

*VPC*, Variance partition coefficient; *PCV*, Proportional change in variance; *MOR*, Median odds ratio; *SE*, Standard error.

Regarding the fixed effect, the results indicated a large provincial difference in childhood undernutrition. As compared with the average level, the odds of childhood undernutrition were higher in Guizhou (OR 2.15, 95% CI: 1.50, 3.08) and Sichuan (OR 1.77, 95% CI: 1.28, 2.45), but were lower in Inner Mongolia (OR 0.41, 95% CI: 0.28, 0.60) and Ningxia (OR 0.49, 95% CI: 0.34, 0.72). It was also noted that older children had a higher prevalence of childhood undernutrition. The boys in the surveyed areas were more likely to become undernourished than the girls (OR 1.27, 95% CI: 1.16, 1.39). Furthermore, there was a higher likelihood of undernutrition in Minority ethnicities in comparison with children from the Han ethnicity (OR 0.77, 95% CI: 0.65, 0.90). In the study, it was also confirmed that mothers’ education levels and wealth index were both inversely associated with childhood undernutrition.

## Discussion

Our study investigated the prevalence and contributing factors underlying undernutrition among children under three years of age in rural Western China in 2005. Children with more than one nutritional deficiency were included in the calculation of stunting, wasting and underweight in the current study. Results showed that the prevalence of stunting as past or chronic malnutrition and wasting as acute malnutrition, were 16.6% and 5.2% respectively, both higher in contrast with other studies in other areas of China. The 2002 China National Nutrition and Health Survey showed that the average rates of undernutrition for children younger than five years old were 7.8% for underweight, 14.3% for stunting and 2.5% for wasting [17]. Another nationwide survey conducted in rural areas of China in 2006 indicated that the rates of stunting, underweight and wasting of children less than 5 years old were 14.59%, 7.19% and 3.07% respectively [18].

As compared to the local data in 1999; however, a significant decrease in prevalence of childhood undernutrition in rural Western China was clearly observed in 2005. The prevalence of stunting in this study was 5.2%, whereas in 1999, it was 7.5%. Hence, the reduction in prevalence from 1999 to 2005 was 2.3% or an annual reduction of 0.4% per year. Similarly, the annual reduction in the prevalence of underweight and wasting were 2.4% and 1.1% per year respectively. At this rate, we speculated that there is a large likelihood of meeting MDG by 2015 in rural Western China.

Globally, the prevalence of acute and chronic malnutrition showed a large diversity. In South Africa in 2007, stunting (18%), wasting (7%), and underweight (10%) were all prevalent for children aged less than 5 years [19]. In 2005 South-central Asia was estimated to have the highest prevalence (16%) of wasting and numbers (29 million). The Eastern and middle Africans have the highest prevalence in estimates of stunting with 50% and 42%, respectively; however, the largest number (74 million) of children affected by stunting lived in south-central Asia [20].

Compared to commonly-used anthropometric indices, CIAF provided a much higher undernutrition rate of 21.7% among children younger than three years old. This rate also varied around the world: 57.9% of undernutrition for children under the age of five in Nepal (2006), 56.1% in Bangladesh (2008), 51.6% in Nigeria (2008), 44.0% in Liberia (2007), 35.6% in Ghana (2008), 36.0% in Egypt (2008), 12.5% in Dominican Republic (2007) [21]. Data presented in the 2006 National Family Health Survey in India showed that 61.8% of the studied children less than five years old suffered from undernutrition [22]. China as a member of BRICS (Brazil, Russia, India, China or South Africa) was recognized as a newly advanced development economy. By comparison, the nutritional status of children in rural western China was better than the counterparts in most mid-low income countries including some BRICS countries such as India and South Africa [19,21,22].

Taking into account the difficulty in comparing variances between individual and area level in multilevel regression, VPC, MOR and PCV were applied to the evaluation of variance at different levels. MOR indicated that in the median case the residual heterogeneity between areas existed when randomly picking out two people in different counties. In comparison with the null model, PCV presented that individual factors in the full model contributed towards a large proportion of the area variance. Controlling for other individual characteristics, approximately 9.8% of variation could be attributed to a cluster effect at the county level by reference to VPC. According to the analysis above, we concluded there was a clear cluster effect in the study areas reflecting that individuals from the same counties tend to be more alike in their background characteristics. Thus, a two-level logistic model was used as the sole suitable method to control for a cluster effect in this study.

The two-level logistic analysis displayed that the great difference in childhood undernutrition among 10 provinces remained even after adjusting for other socio-demographic factors. For example, the likelihood of childhood undernutrition was the highest in Guizhou (OR: 2.15, 95%CI: 1.50, 3.08) and the lowest in Inner Mongolia (OR: 0.41, 95%CI: 0.28, 0.60). There were several possible reasons for the difference between these two provinces. First, Western China represents great diversities in provincial economic level. Among the 10 provinces, the per capita GDP in 2004 was the lowest in Guizhou, which was officially acknowledged as one of the poorest areas in China [23]. Compared to other provinces in Western China, on the contrary, there was a higher per capita GDP in 2004 in Inner Mongolia. Secondly, most participants from Guizhou in the study were of Minority ethnicities with a higher prevalence of childhood undernutrition compared to Han ethnicity. In addition, the two provinces were also different in terms of the percentage of boys (61.8% for Guizhou versus 54.7% for Inner Mongolia, *P <* 0.05), the proportion of children over 6 months (88.6% for Guizhou versus 84.3% for Inner Mongolia, *P <* 0.05) and rate of mothers with no education (15.7% for Guizhou versus 6.3% for Inner Mongolia, *P <* 0.05), all of which had been proved to be positively associated with undernutrition in the present study. Our present findings were also consistent with the studies performed in other areas of China [23,24].

The rate of childhood undernutrition progressively declined with the increment of household wealth. As is well–known, poverty was closely associated with inadequate nutritious food, and poor sanitation and hygiene that were likely to increase the risk for infections and undernutrition in children [25]. Childhood undernutrition was further found to have an inverse correlation with maternal education. As compared with non-educated women, well-educated women tended to have better work opportunities and obtain higher incomes. Accordingly, well-educated women were also more likely to share a higher socioeconomic status in the society and marry with well-educated husbands [26,27]. Previous studies showed that highly educated mothers had adequate health knowledge, lower fertility and more child-centered care, so that they had a better ability to efficiently manage limited household resources and make full use of available health care services to improve child health [28,29].

Childhood undernutrition worsened with increasing age of children. Children in the youngest age group of 0 ~ 6 months had a significantly lower rate of undernutrition than in other older age groups. When based on various classifications of CIAF, it was found the risk for acute undernutrition such as “wasting only” and “wasting and underweight” increased firstly with child age and reached the highest in the 13 ~ 18 months of age and then declined. On the other hand, the prevalence of chronic undernutrition including “stunting”, “underweight and stunting” and “underweight”, increased all the way at ages 25 months and above.

WHO recommends exclusive breastfeeding of infants till 6 months of age and continued breastfeeding until 2 years of age or older [30]. There is evidence available to suggest that breast milk could provide children aged < 6 months of age adequate essential nutrients as well as brings about a lower risk of infection due to the mother’s immune system protection [31,32]. When children grow rapidly with age, breastfeeding alone hardly meets their nutritional needs. Besides mother’s milk, complementary nutritious food should also be included in the child food after the sixth month of age [33]. In rural areas of China; however, most children were given complementary food either earlier or later. Some children at 7 months old or even older had not been given any other food at all, because many mothers believed that breastfeeding alone could provide enough nutrients for their children. Even among children who were given complementary foods, cereal porridge and wheat flour were the only two foods and the children lacked a variety in foods, such as high protein food, fruits and vegetables [33-35]. Studies also showed that in rural areas the intake of meat and eggs as the primary protein sources was very low in the older children. If they do not receive an adequate quantity and quality of complementary foods after the period of exclusive breastfeeding, even children with optimum breastfeeding will first experience acute undernutrition and then chronic undernutrition over time. Therefore, nutritional education was very important and urgent for mothers.

A gender difference in childhood undernutrition was also clearly observed in our study. In accordance with other studies in China, the local boys were more likely to be undernourished than their female counterparts [20,24]. This might be partially attributed to bio-vulnerability of the male gender, but other detailed reasons in study areas required further study. The results also showed the higher odds of childhood undernutrition in the Minority ethnicities versus Han children. Previous studies presented data that approximately 40 million people in Western China remain in poverty nowadays [36]. It is generally estimated that Minorities make up a disproportionally large majority of the poverty population in rural Western China. Moreover, the average education periods of mothers from Minority populations were much shorter than Han mothers in our study (5.9 years vs 7.0 years, *P <* 0.05). All of the above factors might contribute to the differences between different ethnicities.

The strength of our study was the large sample of participants. The introduction of the CIAF also brings more precision to the identification of the more nutritionally vulnerable segment of the population. However, some limitations do exist in our study. First, owing to the cross-sectional design of this study, the causal relationships between socio-demographic characteristics and childhood undernutrition were not confirmed and hence our findings should be interpreted cautiously. Second, the observed differences were subject to many unobserved confounding factors. For example, information on children’s diets and mothers’ nutritional status were not available, and thus could not be controlled in a multivariate analysis. Third, given a lack of blood samples from the children in the survey, we could not determine the micronutrient values and deficiencies of essential vitamins and minerals of the children. Fourth, the group-level chosen (counties or provinces) could be categorized in multiple or overlapping contexts, so it may influence the results.

Due to the lack of information on individual income, the wealth index was adopted to assess individual socioeconomic status through principal component analysis in our study. The objective here was to explore the relationship of socioeconomic status with others factors as a regionally consistent measure of welfare. For reasons of simplicity and interpretation, the classifications of wealth index presented three socioeconomic levels indicating children from the poorest, middle and wealthier household incomes, based on tertiles of the household population. Unfortunately, the percentage of poor individuals defined by wealth index tertiles might be inconsistent with the percentages of poverty reported in the national statistics. Although the use of different cut points for this wealth index was likely to influence results, it was generally a good way of distinguishing between socioeconomic layers within a population in the absence of reliable information on income or expenditures.

Despite these limitations, this study has still provided the most updated information on childhood undernutrition and filled a gap in knowledge of this geographical region.

## Conclusions

In summary, undernutrition among children less than three years old still remains a great health burden in rural Western China. A great difference in childhood undernutrition among 10 provinces was clearly confirmed. Girls of lower than 6 months, Han ethnicity, more mothers’ education years, and wealthier households all benefited child health. Some measures to improve early child development should focus on those provinces with worse economic levels (e.g. Guizhou) and target the older and male children of minority ethnicity by alleviating poverty and enhancing mothers’ nutritional education.

## Competing interests

The authors declare that they have no competing interest.

## Author's contributions

LP and LR designed the prescription study, collected the data, conducted the data analysis and prepared the manuscript; HY contributed to the design and analysis of the study and the preparation of the manuscript. All authors read and approved the final manuscript.

## Pre-publication history

The pre-publication history for this paper can be accessed here:

http://www.biomedcentral.com/1471-2458/14/121/prepub
